# Anomalous variations of VLF sub-ionospheric signal and Mesospheric Ozone prior to 2015 Gorkha Nepal Earthquake

**DOI:** 10.1038/s41598-018-27659-9

**Published:** 2018-06-20

**Authors:** D. V. Phanikumar, Ajeet K. Maurya, Kondapalli Niranjan Kumar, K. Venkatesham, Rajesh Singh, S. Sharma, M. Naja

**Affiliations:** 1 Aryabhatta Research Institute of Observational Sciences (ARIES), Nainital, India; 2 Department of Physics, Doon University, Dehradun, India; 3 Atmosphere and Ocean Research Institute, University of Tokyo, Chiba, Japan; 4 KSK Geomagnetic Research Laboratory, IIG, Allahabad, India; 5 Physical Research Laboratory, Ahmedabad, Gujarat India

## Abstract

The subject of pre-earthquake ionospheric signatures has always been contentious and debatable. Some of the previous reports have documented unforeseen and unusual variations in some of the atmospheric and ionospheric parameters well before an earthquake. Here, we analyze the ionospheric response from the Indian Subcontinent to Nepal Gorkha Earthquakes occurred between April and May 2015, which were the most powerful and disastrous natural calamities in past ~80 years over the Himalayan region left ~9000 causalities and more than ~20000 people injured with the property damage of the order of several billion dollars. In view of severe earthquakes occurrences, their prior information on the shorter time scales are warranted for mitigation of associated disasters. Here, we report for the first time, a case which shows a strong link in anomalous variations between VLF sub-ionospheric signal and mesospheric ozone prior to both April 25, 2015 (M_w_ = 7.8) earthquake and its biggest aftershock on May 12, 2015 (M_w_ = 7.3). Observations show an unusual variation in VLF signals amplitude /shift in terminator time (TT) strongly linked with positive (negative) mesospheric ozone anomaly in D-region altitudes prior to the Gorkha Nepal earthquakes. It is surmised that simultaneous continuous observations of both VLF waves and mesospheric ozone can be considered as an important tool to identify the prior earthquake signatures in the vicinity of the extremely earthquake-prone zone such as Himalayan region. In this context, the current report opens up a new dimension in lithosphere-atmosphere-ionosphere coupling during the earthquake preparation processes itself.

## Introduction

The Indian subcontinent is highly diversified because of its natural geological features. Especially, the young mountainous region of the Himalayan range is highly elevated with the area over ~500000 km^2^ with a high population density (∼200 person km^−2^) and is extremely prone to natural disasters mainly because of meteorological factors and geologically related catastrophes in the form of earthquakes thus making this region imperative to study the causes and damage due to natural calamities. In general, the major types of natural disasters over the Himalayan region can be broadly classified as landslides, forest fires, flash floods, Glacial Lake Outburst Floods (GLOFs) and recurring minor and sometimes major earthquakes. Hence, the Himalayan region is frequently vulnerable to one or more of these types of disasters depending upon the location, local geology, and geomorphology of the region.

Earthquakes (EQs) are considered to be one of the most devastating forms of natural disasters over the globe (causing an approximate economic loss over 75 billion dollars). EQs can cause minor as well as major damage both in terms of property (few billion dollars) and human loss (few thousands) depending upon magnitude, duration, and depth of the earthquake^1^. In this regard, previous studies mainly attempted to identify atmospheric and ionospheric signals prior to some of the major earthquake events on a shorter time scales^2^. Prior information on shorter time scales of the order of few days or a week is the most warrantable way for mitigation of a devastating natural disasters. One of the most important tools which have emerged in the recent past is EQ projection through Electro-Magnetic (EM) method^3^. The EM method utilizes remote sensing techniques to search for change in the lower as well as upper atmospheric abnormal behavior possibly caused by the process of stress buildup and pre-shocks of an impinging EQ. A variety of seismo-physical sources reported till date because of the anomalies of electromagnetic signals associated with the plausible earthquake precursors (e. g., radioactive and thermal emissions, electro-kinetic phenomena). However, the coupling of these physical sources to upper atmosphere from the surface is suggested to happen through acoustic, chemical and electromagnetic channels^4^. The Lithosphere-Atmosphere-Ionosphere Coupling (LAIC) mechanism suggests that the seismic processes start much prior to main earthquake shock and could be seen within the earthquake preparation zone^3^. Since past couple of decades, the scientific community has been trying to understand the existence of reliable anomalies on pre-, co- and post-earthquake scenarios in various ionospheric regions by utilizing electromagnetic signals from ground-based observations and instruments onboard satellites^2,4–8^.

The D-region (50–90 km), the lowest region of the ionosphere, is found to be more responsive to lithospheric disturbances^4,5,9,10^. It is well known that sub-ionospherically propagating Very Low Frequency (VLF) transmitter signals are proved to be the most reliable and cost-effective tool for continuous monitoring of D-region variations due to atmospheric forcing from above/below the ionosphere^11–15^. The VLF signal propagates through multiple reflections between the earth’s surface and lower ionosphere (D-region), which forms a natural waveguide known as an earth-ionosphere waveguide (EIWG)^16^. As the seismo-physical disturbances/variations may directly or indirectly perturb the localized (within preparation zone) ionospheric plasma through lithosphere-atmosphere-ionosphere coupling processes and can significantly ionize/deplete D-region ionosphere^5,9,11,13^.

Reports in the past have illustrated the existence of anomalies in VLF signal few days to even months preceding the EQs using various statistical analysis techniques^2,13^. One of the most important techniques of recent time is the terminator time (TT) method, in which time shift in the two characteristic minima during sunrise and sunset in the diurnal variation of VLF signal prior to an impinging EQ is scrutinized^5,12,13,17,18^. The two characteristic minima (also known as morning and evening terminator) are formed due to the interference of several wave modes during night-day (morning) and day-night (evening) transition periods^15,18^. The diurnal shift in TT is representative of the diurnal day length changes and the conditions associated with the change in the upper boundary i.e. D-region of EIWG^17,19,20^. Therefore, terminator time is a physical quantity that provides useful information on D-region ionospheric changes caused by diurnal conditions or any extreme geophysical phenomena. Several studies in the past convincingly depicted the shift in terminator times before/after the earthquakes^5,18^.

Despite different studies and reports, the pre-earthquake signatures by electromagnetic observations remain a topic of debate due to complications in terms of their morphology. The major problem which still exists is an accurate understanding of how the seismic signal from EQ preparation zone propagates to the ionospheric altitudes that dissipate and cause potential changes in the ionosphere to be identified as prior EQ information. Recent study evidenced a shift in evening terminator time a day before the two major events (M_w_ >  7) of 2015 Nepal Gorkha EQ^13^. This study suggested for finding on any simultaneous ionospheric parameter showing similar anomalous variation and to be convincingly termed as prior EQ signals. Here, we made an attempt to see if any other ionospheric parameter is/are closely linked with anomalous variations seen in the VLF signal (or in the D-region ionosphere). Again, two intense Gorkha EQ events: main EQ of April 25, 2015 (M_w_7.8) and its largest aftershock on May 12, 2015 (M_w_7.3) (hereafter renamed as EQ1 and EQ2 respectively) are considered in order to address the issues and explore the reliability and consistency of prior EQ ionospheric signatures.

In tandem with observed VLF anomaly^13^ during Gorkha Nepal EQ, several simultaneous ionospheric parameters are scrutinized and found that VLF anomaly has a close relation with ozone concentration variation at mesospheric altitudes. Although few studies in the recent past focused on the enhancement of ozone concentration during earthquakes over different parts of the globe by utilizing satellite data along with ground-based observations to better understand the atmospheric variations (columnar ozone, surface temperature, electric field variations) prior to EQs in order to have clarity on the parameters (time, epicentre and magnitude) of the forthcoming earthquakes^21–23^.However, this is the first report to establish a strong relationship between pre-seismic VLF signal amplitude/TT and mesospheric ozone anomalies prior to any major earthquakes over Himalayan region reported till date. Rigorous and detailed analysis is performed with VLF NWC (19.8 kHz) transmitter data recorded at Allahabad (25.41° N, 81.93° E) for two major EQs (EQ1 and EQ2) which occurred within a short span of 20 days apart from the multiple aftershocks subsequent to EQ1 event. In order to have a better contrast and to understand complex underlying EQ processes to identify any other ionospheric parameter having a strong association with observed VLF anomaly, we have investigated further on the responses from both *in-situ* and satellite ionospheric datasets along with lower atmospheric dataset for these two EQs.

**Figure 1:**
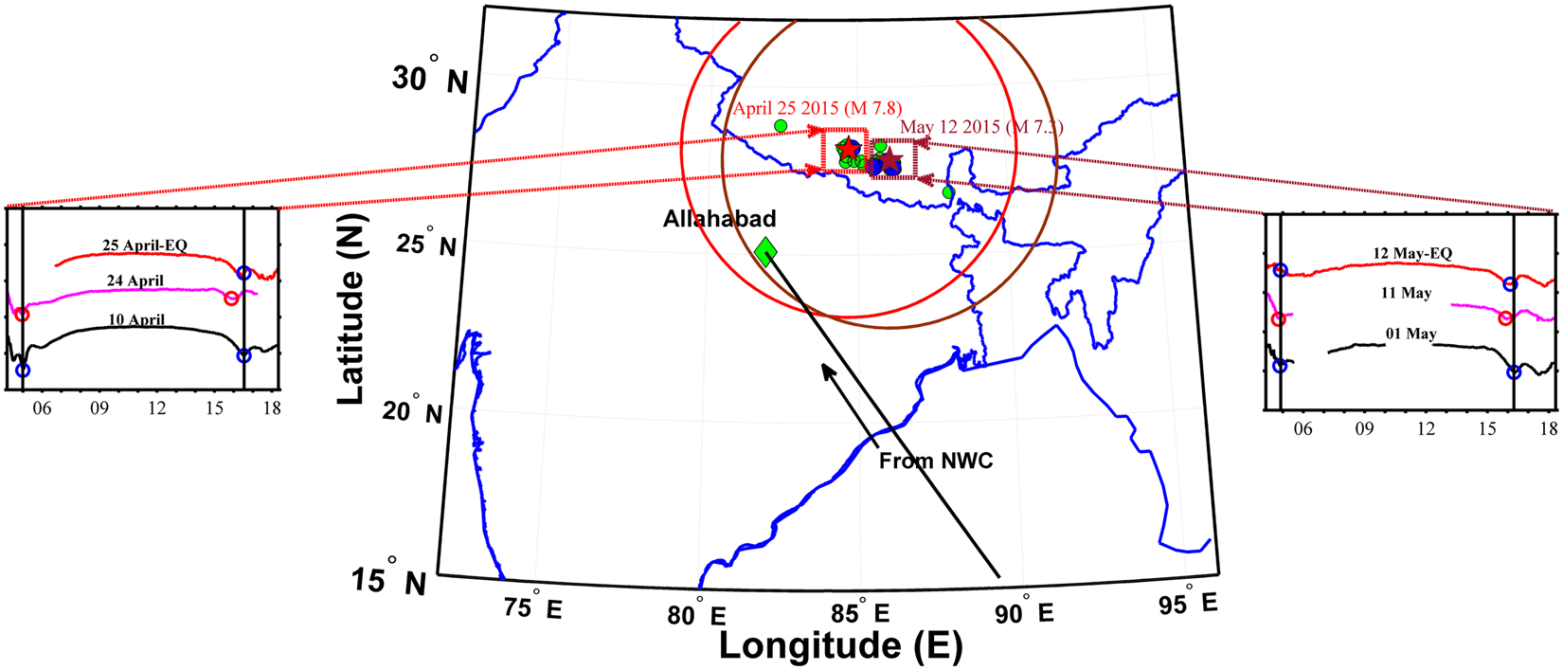
Earthquake locations are depicted during April – May 2015 over Himalayan region highlighting the Gorkha earthquake (April 25, 2015 shown as red star) and a major aftershock (May 12, 2015 shown as brown star) along with VLF transmitting (NWC, Australia) GCP and receiving station (Allahabad). Green (blue) dots represent earthquakes M_w_ > 5.0 (M_w_ > 6.0). Daily Terminator Time (TT) variation for three selected days (10, 24 & 25 - EQ1 of April 2015 and 01, 11, & 12 - EQ2 of May 2015) are plotted in left and right bottom panels, respectively. The resulting map are drawn and encapsulated in a MatLab R2014a figure (https://se.mathworks.com/products/matlab.html).

The prime focus of the present report is to study the atmospheric/ionospheric precursory signatures of the most devastating and long-lasting aftershocks subsequent to April 25, 2015, Gorkha EQ over Nepal and surrounding area in the Himalayan region. The EQ1, the first main shock occurred at 06:11:26 UT (11:41:26 IST) on April 25, 2015, with M_w_ 7.8 at a depth of ~15 km depicted with a red star in Fig. 1. The aftershocks with M_w_ > 5 (M_w_ > 6) is depicted with green (blue) dots respectively. Since the VLF data used is from India, in the analysis the time of observation in Universal Time (UT) has been converted to Indian Standard Time (IST = UT + 5.5 hrs). The EQ1 epicenter was located ~77 km northwest of Kathmandu (28.147° N; 84.708° E). The largest aftershock EQ2 occurred with M_w_ 7.3 and with shallow depth of ~15 km at 07:05:19 UT (12:35:19 IST) on May 12, 2015. The epicenter of this aftershock was located ~75 km east of Kathmandu near Kodari (27.81°N; 86.08°E) is depicted with a brown star in Fig. 1. The study from Allahabad region using VLF observations showed an anomalous terminator time shift and the abnormality observed was attributed possibly to the combination of both chemical, acoustic and gravity waves channels emanated during the major earthquakes of Nepal^13^. Here, we extend this work further and re-analyzed the NWC (19.8 kHz) signal recorded at Allahabad, India (Fig. 1) with a new approach. Daily TT variation of NWC signals for three selected days (10, 24 & 25 - EQ1 of April 2015 and 01, 11, &12 - EQ2 of May 2015) is shown at left and right side of Fig. 1. The blue circle on the daily variations of VLF amplitude represents the normal day TT location during morning and evening period whereas red circles represent the TT location on the April 24 and May 11, 2015, one day prior to both the EQ1 and EQ2 events. The normal day (event day) in the case of 2015 Gorkha EQ1 and EQ2 are 10 April 2015 (EQ1) and 01 May 2015 (EQ2) has been considered after elimination of any effects related to meteorological conditions, and any geomagnetic storm events which may affect ionosphere from below and above^13^. As evident from Fig. 1, evening TT shift with respect to normal day is clearly observed to be ~45 min and ~26 min one day before the main shock in both the EQ1 and EQ2 events on April 24 and May 11, 2015, respectively^13^. However, no noteworthy shift is seen in morning terminator in both the cases. Further, deviation from a monthly mean shifting the evening terminator (~30 min) was observed to be higher for EQ1 (M_w_ 7.8) than TT shift in evening terminator (~14 min) for EQ2 (M_w_ 7.3).

**Figure 2:**
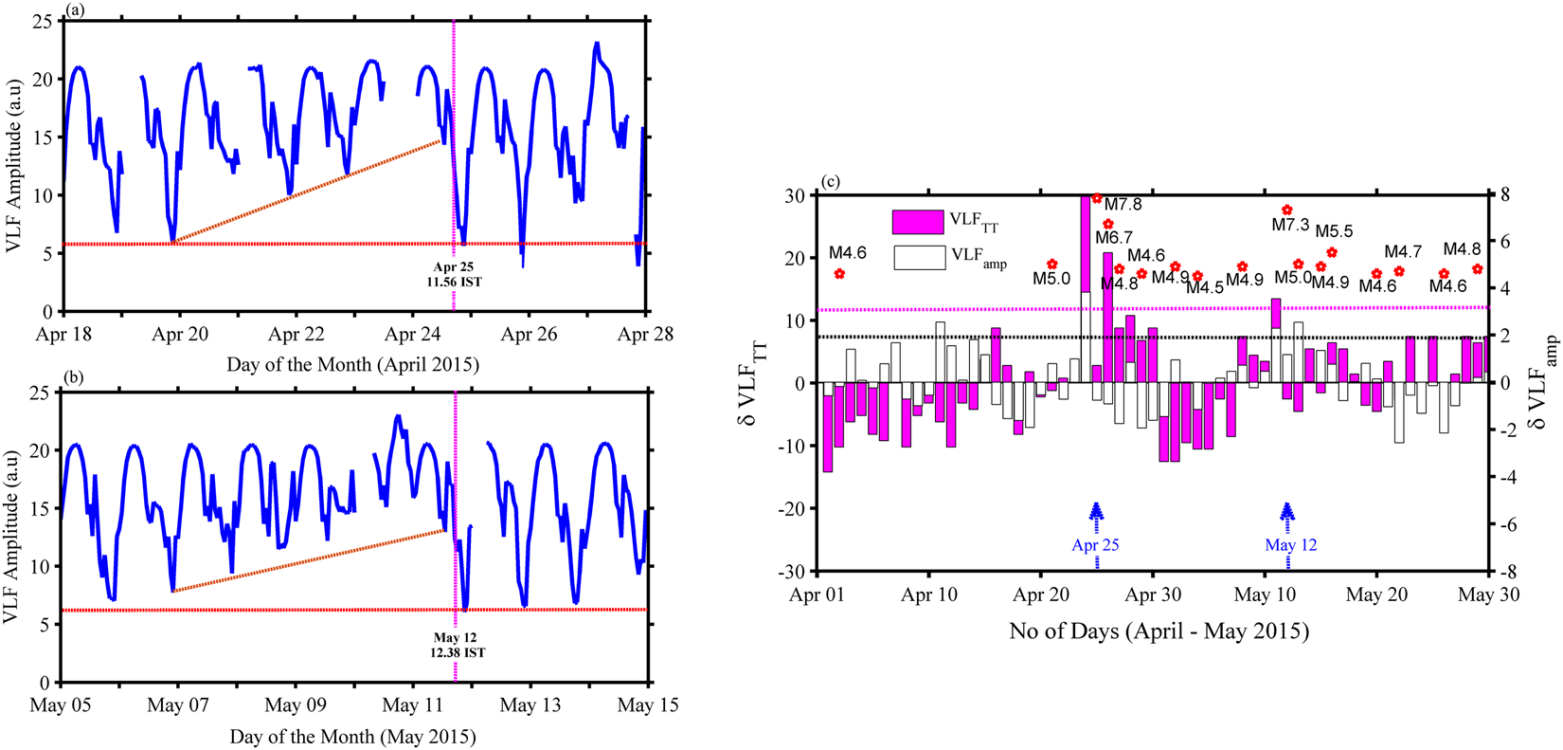
Time series of δVLF_amp_ (white bars) over plotted with daily evening δVLF terminator time (TT) shift (magenta bars) and corresponding earthquakes with magnitude (M_w_ > 4.0) for the complete months of April and May 2015. Horizontal lines represent 2σ (95% confidence) level for both VLF amplitudes (black line) and TT shift (magenta line), respectively. Vertical magenta line represents both the EQ1 and EQ2 days.

Figure 2a,b depicts the temporal evolution of VLF amplitude for both the EQ cases (April 18–28 & May 05–15, 2015). It is interesting to note that an anomalous shift exists in the terminator as well as nighttime amplitude variation (shift is towards higher amplitude end) in a systematic manner during both the EQs although major solar activity was not observed during the observational period. In another way, we can say that there is a systematic input into the D-region from some external source other than the solar origin. It is to be noted here that there was neither strong lightning activity nor some additional input from extra-terrestrial sources, indicating that the source could be down below (lower atmospheric origin) which has significantly contributed in modifying the D-region chemistry and dynamics^13^. Figure 2c represents δVLF_amp_ time series (white bars) overplotted with daily evening terminator time (δVLF_TT_) shift (magenta bars) and corresponding earthquakes with magnitude (M > 4.0) for the complete months of April and May 2015. Magenta (Black) dotted lines indicate 2σ level (95% confidence level) to identify anomalous TT shift (corresponding to VLF amplitude) and EQ magnitude with statistical significance. The time series measured here for δVLF_amp_ signal amplitude (a.u) is during evening terminator for two hours of 15:00–17:00 IST (IST = UT + 5.5 hrs) to consider amplitude variation during evening TT period only. It is to be noted that both δVLF_amp_ signal amplitude and the δVLF_TT_ shift is maximum and crossing 2σ level on April 24, 2015 (May 11, 2015) one day prior to both the major earthquakes (EQ1 and EQ2). One important and striking feature noted here is that δVLF_amp_ signal amplitude and δVLF_TT_ TT shift are in-phase on both the days prior to both the EQs whereas out of phase in all other cases although occasional peaks are observed in both the parameters with respect to multiple aftershocks following the major EQ1 during this two months period.

To better understand the NWC signal variations and D-region ionospheric response during Gorkha earthquake, Long Wave Propagation Capability (LWPC) modeling code^24^ was used on the NWC VLF signal for both the events of EQ1 & EQ2. The Wait’s model of the lower ionosphere is characterized by two parameters: the sharpness (β in km^−1^) and reflection height (H′ in km)^25^. The modeling results (H′ and β values) for EQ1 and EQ2 event days and normal days are presented in Table 1 (details about the LWPC model has been provided in methodology section).

**Table 1:** The H′ and β values modeling results for EQ1 and EQ2 event days and normal days.

Event (s)/Parameters	Normal day 10 Apr (01 May) 2015	Day prior to EQ1 (EQ2) 24 Apr (11 May) 2015	EQ1 (EQ2) day 25 Apr (12 May) 2015
Amplitude (dB)	48.88 (51.35)	54.34 (53.00)	50.12 (52.02)
H′ (km)	82.5 (82.09)	78.30 (79.70)	81.90 (81.50)
β (km^−1^)	0.28 (0.27)	0.2900 (0.28)	0.2800 (0.28)

From Table 1, ionospheric reflection height (H′) shows a maximum decrease of 4.2 km and 2.39 km on one day prior to both EQ1 and EQ2 when compared to their respective normal days. The change in the values of H′ and β are directly related to change in the D-region electron density (ED)^25^. H′ decrease or lowering of D-region bottom boundary represents an increase in D-region electron density one day prior to both EQs. On main EQ days of 25 April and 12 May, the difference in H′ in comparison to normal day was reduced to 0.6 km and 0.59 km and this indicates that the D-region density is recovered on both main days of EQ1 and EQ2.

**Figure 3:**
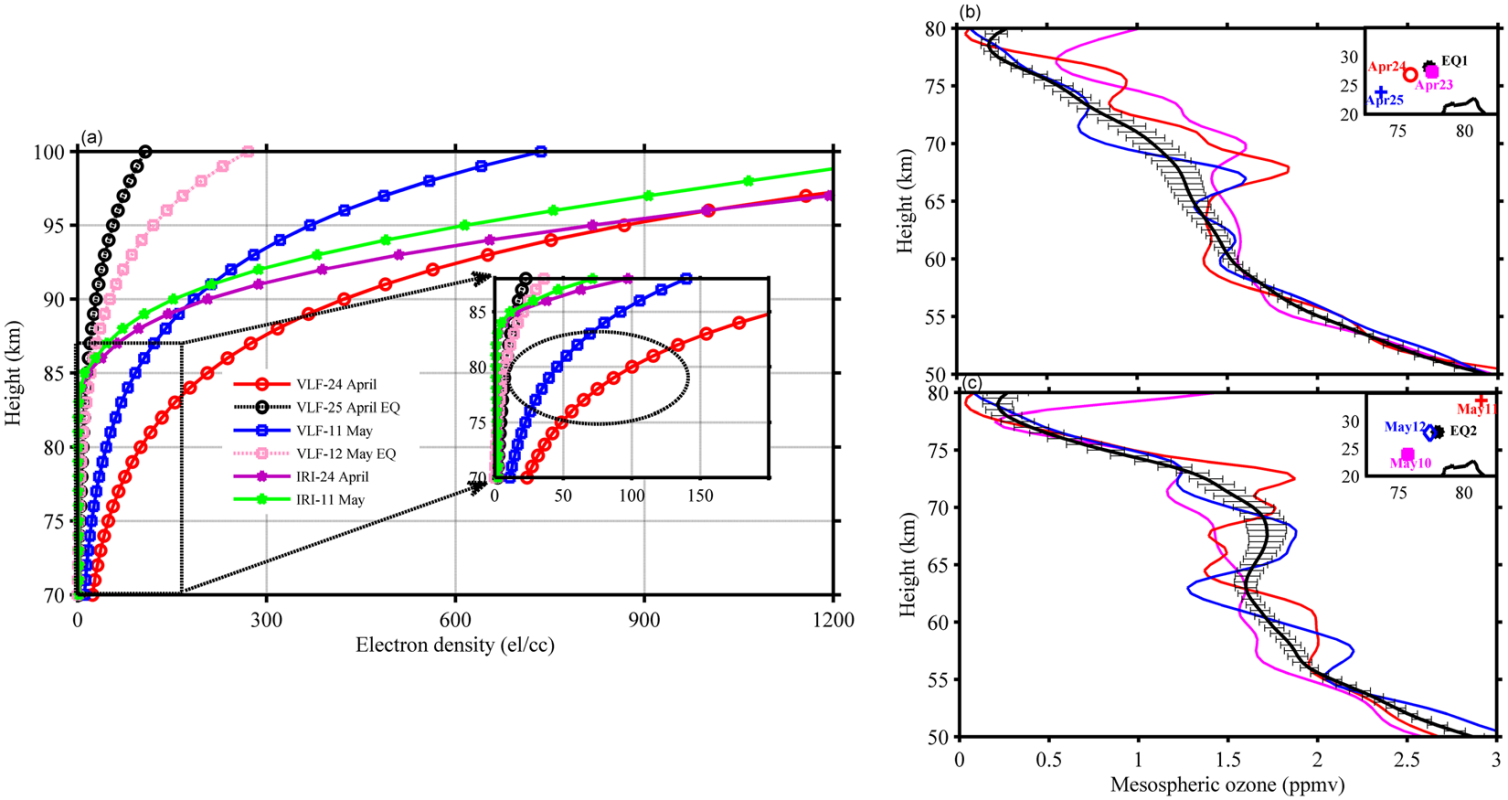
(**a**) Vertical profile of electron density estimated from LWPC model by using VLF data for Gorkha EQ cases (April 24–25; May 11–12 2015) along with IRI model Electron densities during April 24 and May 11, 2015 (a day before main EQs). Zoomed version of the figure is shown in the inset to depict the minute variations in the electron densities in D-region heights. (**b**) and (**c**) Illustrates the vertical profiles of mesospheric ozone for both the EQ cases (April 23–25; May 10–12 2015) along with monthly mean ± 2σ curve. The inset shows the SABER satellite pass with respect to the epicenter of respective EQ cases.

It is also important to note that highest δVLF TT shift, δVLF amplitude and maximum decrease in H’ (Fig. 2 and Table 1) occurred one day prior to both major shocks of EQ1 and EQ2. This implies an increase in D-region electron density one day prior to both EQ1 and EQ2. Hence, in order to understand qualitatively the variations in the electron density in the D-region altitudes (70–90 km), vertical profiles of electron density calculated utilizing H′ and β parameters before/after EQs^24,25^. are presented in Fig. 3 (left panel inset) along with SABER ozone anomaly averaged from heights 50–80 km (right panel). Here, we introduce the mesospheric ozone parameter as this was the only parameter which showed a close connection with an observed anomaly in VLF observations. Electron density profiles obtained from the IRI-2016 model on April 24, and May 11, 2015 are also incorporated for comparison in Fig. 3 (left panel). IRI electron density has been estimated at EQ epicenter location for respective EQs (EQ1 and EQ2) at 11 UT (~16:30 IST, evening terminator time). As evident from the Fig. 3 (left panel inset), a four-fold increase in the ED of the order of ~206 el/cc and ~92 el/cc at 85 km altitude was observed on a day before both the EQ1 and EQ2 on April 24, 2015, and May 11, 2015. Interestingly, the decay in the electron density is directly linked with the magnitude of the earthquake, depicting higher values one day prior to EQ1 and subsequent rapid decay on the day of EQ1 (M7.8). However, the major after-shock EQ2 (M7.3) of 12 May event had relatively less rapid decay in ED as compared to the main EQ1 event. Hence, this shows that intense magnitude EQs manifests in higher electron density but rapid decay. Moreover, a comparison of LWPC derived ED with theIRI-2016 model is made for both EQ1 and EQ2 events. It is interesting to note that ED profiles in lower ionospheric attitudes (60–85 km) are matching with normal days, however, at higher ionospheric altitudes (above 85 km) clearly matching with LWPC model. It should be noted that smaller but minor differences are always there when dealing with two different models whose input parameters vary significantly (IRI model does not provide reliable and accurate D-region parameters (ED)). IRI model not only shows a change in electron density in higher altitudes but also increases rapidly with altitude thereafter, however, ED from LWPC model show low and steady increase. Additional vital point is that both models show low ED during anormal day for EQ2 as compared to EQ1 which is a consistent pattern from LWPC as well as IRI models.

After scrutinizing ionospheric parameters of D-region, mesospheric ozone in the altitude range of 50–80 km was found to be closely associated with the D-region electron density which we discuss further and suggest that observations of VLF anomaly along with mesospheric ozone in future may serve as a potential tool for identifying an impinging earthquake on the shorter timescales. To understand the contribution of D-region dynamics and chemistry, vertical profiles of Sounding of the Atmosphere using Broadband Emission Radiometry (SABER) ozone in lower ionospheric levels (50–80 km) were chosen. Figure 3 (right panel) shows the vertical profiles of SABER ozone centering the epicenter of both the EQ1 (top of right panel) and EQ2 (bottom of right panel) cases during 23–25 April and 10–12 May 2015. The SABER paths considered for the present analysis are shown as red circles in the inset of Fig. 3 (right panel) near to the epicenter, depicting the nearest data point chosen within that circle of influence (~5°) irrespective of day and nighttime. It is clearly evident from both the figures (right panel) that mesospheric ozone anomaly is showing clear and remarkable depletion (enhancement) in lower (upper) altitude regions from the monthly mean (solid black curve represents mean ± 2σ). Vertical profile of mesospheric ozone, a day prior to EQ is completely disturbed with some oscillatory pattern, however, depending upon magnitude, location, and depth of EQ event in both the cases. Mesospheric ozone percentage of variation estimated a day prior to EQ1 (EQ2) i.e., April 24, 2015 (May 11, 2015) is of the order of 30–40% (20–30%) in the altitude region (55–75 km) where statistically significant variation is clearly observed. Few reports in the past tried to understand mesospheric ozone variations with few event-based studies of geomagnetic storm and solar eclipse etc^26–28^ and evidenced significant discrepancies (upto even 30%) in 70–80 km altitude regions. However, the variation of mesospheric ozone is complicated in lower/upper altitudes where ion chemistry alone is sufficient/not sufficient to explain the observed discrepancies. Moreover, as per author’s knowledge, no reports are available till date on the association between D-region electron density and mesospheric ozone pre-, during and post-earthquake events.

It is evident from previous reports that the mesospheric ozone has significant diurnal variability with maximum (minimum) during nighttime (daytime), respectively^29^. Hence, in order to have simultaneous profiles of both mesospheric ozone and VLF (terminator time calculated ± 2 hours from the time of sunset), we have analyzed SABER observations of mesospheric ozone. The SABER observations are taken mostly during daytime hours (10–18 hours local solar timings). The data chosen after the timing scrutiny qualify the conditions from Day No. 110–133 (total of 34 continuous profiles), fortunately covering both the events with the dates constituting from April 20–May 13 2015 (SABER profile details are provided in supplementary material as Table T1). Other ozone profiles during April–May 2015 are of nighttime and hence are not considered because of significant variability. One of the most important contributors to the mesospheric ozone variability is the contribution of internal gravity waves. First, the anomaly is calculated for all the selected profiles (by subtracting mean from each individual profiles). In order to remove the gravity wave contribution, each individual profile is subjected to low pass filter with a cut-off vertical wavelength of 6 km. This vertical wavelength limit is chosen based on the maximum vertical wavelength due to gravity wave fluctuations observed in ozone profiles. However, it should be noted that contribution from acoustic-gravity waves (higher vertical wavelengths ~20 km) may still be present as these may have generated from seismic processes as suggested by some of the previous reports^30–32^.

**Figure 4:**
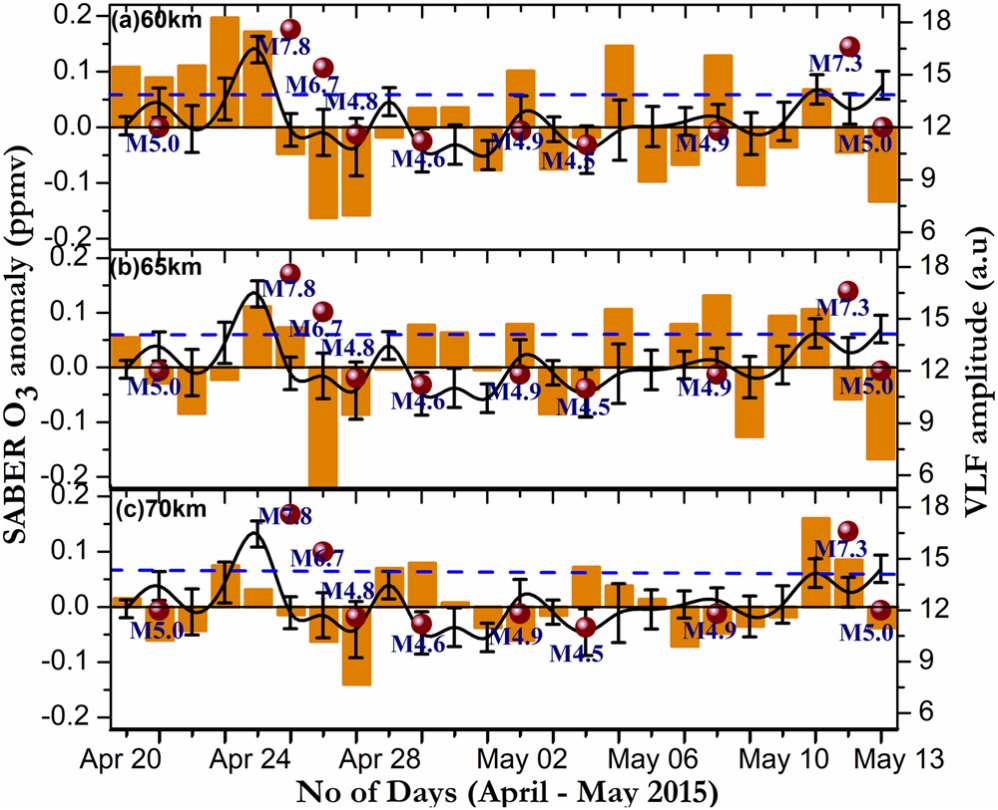
Temporal variation of SABER mesospheric ozone anomaly along with mean VLF amplitude (spline interpolated solid black line) and magnitude of EQs (black circles) for three different regions (**a**) 60 km, (**b**) 65 km and (**c**) 70 km, respectively during April 20 – May 13 2015. Blue dotted line shows 95% significance level.

Figure 4a–c illustrates the temporal variation of mesospheric ozone anomaly along with VLF amplitude variations for three height regions (60, 65 and 70 km) during April 20 – May 13, 2015, over the region of interest. It is clearly evident that strong and systematic mesospheric ozone (greater than 2σ level) and VLF amplitude enhancement prior to EQ1 (April 22–24 2015) and EQ2 (May 10–11 2015) in all the height regions suggesting an increase of ozone concentration prior to the earthquake. It is interesting to note that strongest negative anomaly and a corresponding decrease of VLF amplitude exists especially during major EQ cases (Mw > 6) where we have two strong EQ cases within two days (April 25 & 26 2015) and also during the EQ2 case (May 12, 2015). However, mesospheric ozone pattern seems to be random in nature for some of the minor EQ cases in day-to-day variability due to multiple aftershocks subsequent to EQ1 along with local and regional factors governed. Moreover, location and local solar time of the satellite pass also play a vital role in the lower ionospheric dynamics. The observed variability of mesospheric ozone anomaly decreasing (increasing) with increasing (decreasing) altitudes (above/below 70 km) signifying the role of ionospheric electrodynamics along with ion chemistry through atomic oxygen as we go to higher altitude regions in governing the D-region dynamics^21–23,33^.

Our results depict that both VLF parameters (TT/ED) and ozone anomaly are undergoing similar and noteworthy variations/fluctuations in the mesospheric heights implying that the source for these changes may be related to common phenomena, which is an earthquake in the present case. This anomalous association of D-region ED and mesospheric ozone can be understood as this region of the ionosphere is closely linked with ozone photochemistry and corresponding variations in ionospheric electrodynamics. Increase in mesospheric ozone corresponds to increase in the D-region electron density and hence will have an enormous effect on the propagation of radio waves at very low frequencies whose reflection heights fall in ~60–90 km height region. Hence, results show a strong link between mesospheric ozone and VLF signal anomaly along with the support indirectly by the wave modeling results of previous works^17,20^ suggesting that the TT shift could be associated with 1-2 km decrease in upper boundary of EIWG (D-region reflection height).

The results of association of VLF waves and mesospheric ozone observations during April–May 2015 Nepal EQ clearly depict enhancement in D-region electron density with a systematic increase during a 5-day period having maximum one day prior to both the earthquakes. VLF observations were also found to have an interesting link with anomalous increase/decrease of mesospheric ozone in different altitude regions. In general terminology, variation in mesospheric ozone signifies the variation in electron density especially at lower ionospheric heights (happens to be in the VLF reflection region) as shown in the previous reports^34–37^. The observations from both ground-based (VLF) and satellite datasets (SABER) along with D-region ionosphere modeling results for the first time provided tangible support for their simultaneous monitoring in the studies of pre-earthquake ionospheric signatures. In general, earthquakes are considered to affect the ionospheric electrodynamics through the generation of electric and magnetic fields because of intense seismic activity triggered (crack formation and crustal deformation, fault failure induced magnetism, stress and related conductivity variations etc.) during the earthquake preparation processes itself ^36,37^. The enormous increase of ground radioactivity (especially radon increase in the seismically active zone) may be the primary cause of all atmospheric and ionospheric anomalies observed prior to earthquakes by different groups in the recent past. Further, variations in air conductivity leading to modifications in the global electric circuit may induce the magnetospheric/ionospheric perturbation electric fields thereby resulting in the precipitation of high energetic particles, especially in the lower D-region ionosphere. The precipitation of high energetic particles could probably alter the mesospheric ozone concentration thereby leading to modification in VLF amplitudes/TT/electron density prior to earthquakes^36,37^. However, positive/negative anomalies prior/during the EQ of mesospheric ozone (corresponding VLF amplitude/TT/electron density) as evidenced in the present report (Figs 2–4) can be interpreted considering the relatively shorter life time of mesospheric ozone (few secs to less than an hour)^28,38^. In this context, Lithosphere-Atmosphere-Ionospheric Coupling (LAIC) model provides a better understanding of the physical processes involved in understanding the short-term variations in surface, lower atmospheric and ionospheric pre-seismic signatures before the major earthquakes which can derive from the observed anomalies in different atmospheric/ionospheric parameters within the earthquake preparation process^36,37^. On the other hand, significant contribution from acoustic-gravity waves (higher vertical wavelengths ~20 km) also cannot be neglected considering the generation of these waves during intense seismic activity as suggested by some of the previous reports^30–32^. In the view of the above, more coordinated efforts along with the continuous monitoring of upper atmospheric parameters in different altitude regions could be a valuable input to the scientific community to understand the complex lithosphere-atmospheric coupling during the EQ preparation process and much well before the Major EQs hit the surface of the earth thus resulting in devastating consequences to both human life and property.

## Methods

### VLF data analysis

The VLF data as shown in Figs 1–3 are from the site of Allahabad (25.4°N, 81.9°E) recorded by AWESOME VLF receiver. The AWESOME receiver is setup by Indian Institute of Geomagnetism, India in collaboration with Stanford University, USA under International Heliophysical Year 2007/United Nations Basic Space Science Initiative (UNBSSI) program^39^. The station is located at Dr. K. S. Krishnan Geomagnetic Research Laboratory, Allahabad (Geog. lat., 25.4°N; Geog. long. 81.93°E) (a regional center of Indian Institute of Geomagnetism, Navi Mumbai). The receiver is capable of recording two types of data: broadband data (300 Hz to 47.5 kHz) and narrowband data (fixed frequency VLF transmitter). The recording system consists of two orthogonal crossed loop antennas aligned in North-South and the East-West magnetic planes, with matched pre-amplifier and a line receiver. The pre-amplifier is kept near the receiving antenna feds amplified data to the line receiver by a long cable. Time synchronization is achieved by the GPS clock connected to the line receiver and the data is recorded at 100 kHz, 16-bit sampling rate, and 10 micro-seconds time resolution. The narrowband data is recorded with a sampling of 50 Hz. Here, we have used hourly averaged data for the present analysis. The collected data are analyzed by codes developed in MATLAB.

### LWPC model

LWPC modeling is most reliable code for VLF propagation studies. The LWPC model is a versatile code developed by US Navy to model VLF wave propagation through EIWG^24^. LWPC accepts themodel solution of wave propagation, which treats earth-ionosphere waveguide as a parallel plate, with the ground as conductor and D-region ionosphere as magnetized collisional plasma. This code divides TRGCP into a segment of equal sizes. The wave electric field is calculated sequentially at each segment with the user-defined ground conductivity and permittivity.

The LWPC code realizes lower ionosphere (D-region) as Wait ionosphere. The Wait ionosphere is characterized by two parameters reference height (*H*′ in km) and sharpness factor (*β* in km^−1^) which are being further used to estimate model electron density (ED) profile up to 100 km using wait’s equation^40^. The model electron density is given by the following equation:

1 $$Ne(h)=1.43\times {10}^{7}[\exp (\,-\,0.15\,h)\exp (\beta -0.15)(h-H^{\prime} )]c{m}^{-3}$$

Where *Ne(h)* is the model electron density as a function of altitude *h*.

We have modeled VLF signal using LWPC by choosing a various set of H′ and *β* pairs for given time and transmitter-receiver path length. Moreover, it requires simultaneous measurements of both amplitude and phase, then one needs to run LWPC at the desired time for a various set of h′ and beta values. Each time when we run LWPC, we will get amplitude and phase, so we have to choose a set of h′ and beta which provides observed amplitude and phase at a given time. This method could be used at any time of day or night or event at the terminator. Further, we have noted down the set of *H*′ and *β* values for which LWPC modeling results compare to observations (VLF signal at given time and given TRGCP). Then we get the unique value of H′ and *β* pairs. However, for the first time, LWPC modeling on the VLF signal is used to understand the variations in electron density prior to both the EQ events (EQ1 & EQ2). A sample diurnal profile of VLF amplitude (observed and calculated) on a control day is provided as supplementary figure (Figure S1).

We have used terminator time (TT) method as discussed by many investigators in the past^5^. The terminator is the characteristic minima near the sunrise and sunset of local time generated due to the modal interference between different VLF signal modes^11,13^. In the TT method, attention is paid to the time of morning and evening terminator and time shifts are analyzed near the TT before and after the earthquakes. It is also observed that ET is more pronounced for EQ related changes^5,13^. In the present analysis, we have estimated TT (ET) in the following way. First, we have estimated ET time on 01 April 2015 and 01 May 2015 and considered this as reference TT time. Then the estimated ET time on each day of April and May months are subtracted with the reference time of each month, respectively. This provides with ET shift (in minutes) for each day with reference to a reference day of each month.

### SABER data analysis

SABER is one of the main instruments onboard Thermosphere, Ionosphere, Mesosphere Energetics, and Dynamics (TIMED) satellite that measures vital chemical species in the upper atmosphere. Out of several species, the ozone is the key element measured by SABER in the Mesosphere and Lower Thermosphere (MLT) region for understanding the energy balance. The ozone is measured in two different spectral channels; one at 9.6 µm and the other at 1.27 µm. The data from the 9.6 µm ozone is accessible at all local times while the 1.27 µm ozone is available only during daytime hours. In this study, we use ozone retrieved from the 9.6 µm spectral channel during the evolution of EQ. The peak 9.6 µm emission is measured within a spectral band from 9.9 µm to 8.7 µm to highlight the emission measurement from the fundamental (001-000) band in the asymmetric stretch (ν_3_) mode of ozone^41,42^. The temperature and pressures derived from the SABER observations are also used in the ozone retrieval^43^. A sample vertical profile of mesospheric ozone on a control day is provided as supplementary figure (Figure S2).

### Data availability

The VLF magnetic field recordings that support the findings of this study are available from author RS, but restrictions apply to the availability of these data as they are proprietary of the institute, and so are not publicly available. However, data are available upon reasonable request from the author for the collaborative scientific and research purposes. Other datasets used in the study are available in public domain.

## Electronic supplementary material

Supplementary Figures and Table
