# Supplementary material for: Anomalous variations of VLF sub-ionospheric signal and Mesospheric Ozone prior to 2015 Gorkha Nepal Earthquake
Source: Sci Rep. 2018 Jun 20;8:9381. doi: 10.1038/s41598-018-27659-9 (PMC6010448; doi:10.1038/s41598-018-27659-9)
Supplement: Supplementary file 1 — Supplementary Figures and Table [file 41598_2018_27659_MOESM1_ESM.doc]

**Anomalous variations of VLF sub-ionospheric signal and Mesospheric Ozone prior to 2015 Gorkha Nepal Earthquake**

**D. V. Phanikumar^1^*, Ajeet K Maurya^2^, Kondapalli Niranjan Kumar^3^, K Venkatesham^4^, Rajesh Singh^4^, S Sharma^5^ & M Naja^1^**

^1^Aryabhatta Research Institute of Observational Sciences (ARIES), Nainital, India

^2^Department of Physics, Doon University, Dehradun, India

^3^Atmosphere and Ocean Research Institute, University of Tokyo, Chiba, Japan

^4^KSK Geomagnetic Research Laboratory, IIG, Allahabad, India

^5^Physical Research Laboratory, Ahmedabad, Gujarat, India.

^*^Correspondence to: astrophani@gmail.com

**Figure S1:** A sample output from LWPC model (observed and calculated) on a control day

**
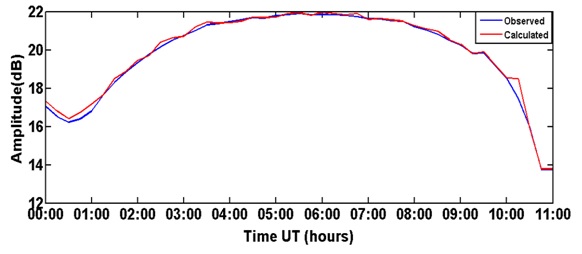
**

**Figure S2:** A sample vertical profile of SABER mesospheric ozone on a control day

**
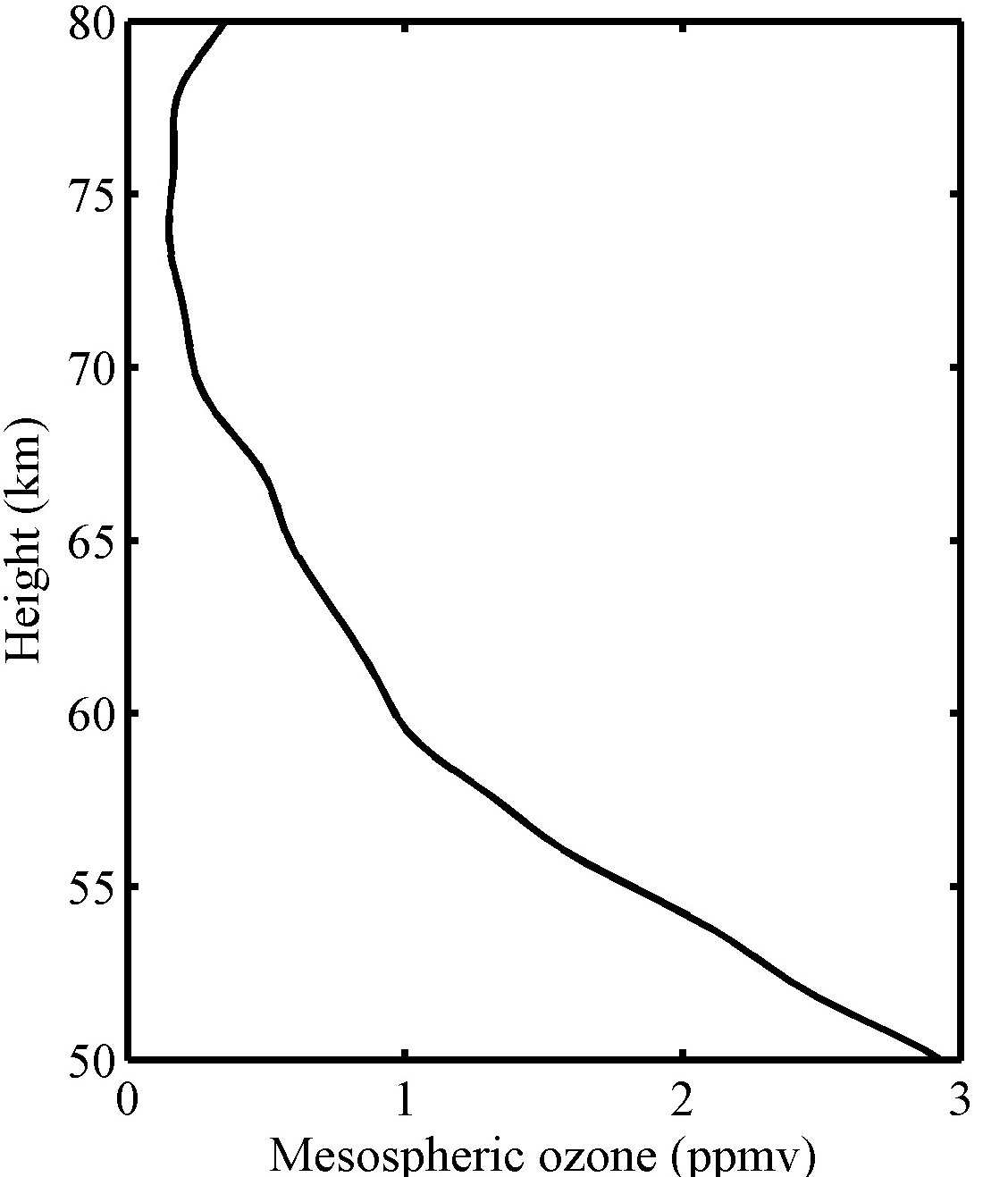
**

**Table1: List of dates of passage of SABER over the observational region/period used in the present study**

| **Date** | **Time (UT)** |
| --- | --- |
| 20-04-2015 | 18:16:0 |
| 21-04-2015 | 18:21:0 |
| 22-04-2015 | 17:33:0 |
| 23-04-2015 | 17:31:0 |
| 24-04-2015 | 17:34:0 |
| 25-04-2015 | 17:29:0 |
| 26-04-2015 | 16:49:0 |
| 27-04-2015 | 16:51:0 |
| 28-04-2015 | 16:41:0 |
| 29-04-2015 | 16:47:0 |
| 30-04-2015 | 16:02:0 |
| 01-05-2015 | 16:01:0 |
| 02-05-2015 | 15:55:0 |
| 03-05-2015 | 15:28:0 |
| 04-05-2015 | 15:20:0 |
| 05-05-2015 | 15:13:0 |
| 06-05-2015 | 15:14:0 |
| 07-05-2015 | 14:39:0 |
| 08-05-2015 | 14:30:0 |
| 09-05-2015 | 14:28:0 |
| 10-05-2015 | 14:29:0 |
| 11-05-2015 | 13:51:0 |
| 12-04-2015 | 10:14:0 |
| 13-05-2015 | 10:02:0 |
